# A novel intra-tumoral drug delivery carrier for treatment of oral squamous cell carcinoma

**DOI:** 10.1038/s41598-023-38230-6

**Published:** 2023-07-25

**Authors:** Shimaa A. Elsaady, Moustafa N. Aboushelib, Essam Al-Wakeel, Manal F. Badawi

**Affiliations:** 1 Dental Biomaterials, Faculty of Dentistry, Zagazig University, Mansoura, Egypt; 2 Dental Biomaterials, Faculty of Dentistry, Alexandria University, Alexandria, Egypt; 3 Dental Biomaterials, Faculty of Dentistry, Mansoura University, Mansoura, Egypt

**Keywords:** Cancer, Drug discovery

## Abstract

The treatment of oral squamous cell carcinoma (OSCC) includes systemic chemotherapy and is associated with aggressive side effects on patients. This study evaluated a new intra-tumor-targeted drug delivery method for the treatment of OSCC induced on the dorsum of the tongue in white mice. The induced tumors were examined by needle biopsy. A targeted anticancer drug (Cetuximab) and [Cisplatin and 5 Fluorouracil (5-FU)] chemotherapeutic agents were loaded on polyethylene glycol-polylactide-polyethylene glycol (PEG-PLA-PEG) nanoparticles (NPs) designed for intralesional injection while systemic administration was used as control. Fourier transform infrared spectroscopy (FTIR) was performed to study NP chemical structure, a drug release profile was conducted to study release kinetics, and histopathological evaluation was performed before and after treatment to evaluate tissue reactions (n-28, ά = 0.05). The drug release profile was characteristic of the chemotherapeutic agent showing early quick ascend followed by sustained slow release. FTIR peaks identified the polymeric structure of the drug nano-carrier. Histopathologic examination of chemically induced OSCC revealed different grades ranging from non-invasive to invasive stages of OSCC. Intra-tumoral test group revealed significant remission of observed cancer grade compared to the systemically administered group (X^2^ = 12.63, *P* < 0.001). Finally, using synthesized PEG–PLA–PEG NPs for intralesional injection is a promising route for the treatment of OSCC.

## Introduction

New updates in medicine have been raging a war on cancer for a long time and despite great improvement in this field, cancer still devastates a lot of lives each year^1^. Aggressive chemotherapeutic agents have drastic side effects on the patients^2,3^. Following systemic application, the treatments before reaching maximal therapeutic effect at the target tissue, they must pass through complex environments and biological barriers as passing through the systemic vasculature, absorption, and retention within the epithelium, diffusion from blood vessels to the underneath tissue, and perfusion through the spaces between the epithelium and the basement membrane. Uncontrolled drug concentrations at the tumor site resulted in the occurrence of multiple drug resistance and the appearance of unfavorable side effects including cancer cell resistance to chemotherapeutic drugs^4^.

Some chemotherapies like 5-fluorouracil (5-FU) and/or gene-targeted treatment as epidermal growth factor receptor can be administered with platinum-based therapy to achieve rapid and intended favorable progress within a short time as a baseline treatment for many types of tumors^5,6^. Conventional therapeutic routes require enhancement in targeted delivery to the lesion site to unmet the unneeded side effects of the drugs. Nanotechnology is currently gaining attention as a therapeutic approach that is no longer novel. It can be achieved through the application of triggered drug release, multi-modal therapies, specific targeting, novel routes of application, and combination^7^.

One application of this technological revolution is nanoparticle (NPs) loaded medication. It is an alternative way of reducing systemic drug toxicity^4^.

This tissue intoxication arising from the organized hinders that hamper the drug delivery to its selected site when the drugs are applied systematically^8^. Drug carriers in numerous forms like drug conjugates, nano-capsules, and micellar systems have been proposed. NPs are nano-sized colloidal particles, where the therapeutic agent is loaded within the particle–matrix, and enhance drug distribution, efficacy, and stability^9^. Due to their unique properties, nanoparticles (NPs) are being used to deliver chemotherapies to cancer cells^10^. Furthermore, the main feature of NPs is their capacity to target specific tumor tissues and release their content in a specific fashion to achieve the maximum therapeutic effect with minimum side effects^11^.

Intra-lesional route of application was one of the approaches to local drug delivery. The principal benefit was that drug exposure was restricted to the lesion with little or no systemic drug exposure. Intraoral targeted chemoprevention via local drug delivery using polymeric systems positively inhibited OSCC outcomes in two aspects: interfering with the progression of premalignant lesions to OSCC and impacting tumor recurrence in previously treated patients^12^.

Among different materials, the FDA has already approved a few drug delivery systems which are based on approved polymers such as polyethylene glycol (PEG)^10,13^. Coating intravenous administered polymeric NPs by PEG prevents them from being engulfed by the macrophages. Coating the hydrophobic PLA with hydrophilic PEG resulted in the copolymerization of both^14^. Designed PLA-based drug delivery systems showed sustained release profiles for the drugs used for low soluble drug delivery as they can be loaded with both hydrophobic and hydrophilic treatments^15^.

Thanks to their special core/shell structures yield they are one of the most promising platforms for drug delivery. In addition, it was determined that an increase of hydrophilicity in nano-micelles (reduction of PLA relative to PEG) played a significant role in the stability of anticancer drugs and release profile with their lower initial burst release, sustained, and slow release of anticancer drugs with minimum drug dosage^16^.

Cisplatin-loaded polymeric micelles were prepared, and PEG-poly glutamic acid block copolymers were applied as a tumor-targeted drug delivery system. These micelles manifested prolonged blood circulation 20-fold higher than drug-free micelles. It also had shown complete tumor regression with no significant body weight loss. Four of 10 mice treated with CDDP/m (4 mg/kg; two days 2-day intervals, five times administration) manifested complete tumor regression with no significant body weight loss, whereas treatment with the same regimen and drug dose of free CDDP resulted in tumor survival and ∼20% of body weight loss. These micelles manifested prolonged blood circulation and remarkably accumulated in solid tumors^17^.

In the xenograft mouse model, tumor-targeted PEG-PLA NPs were synthesized for the delivery of cetuximab, 5-FU, and radionuclide iodine-131. It was found that these loaded NPs exhibited prolonged circulation in the blood and accumulation in the lesion site, thus revealing improved antitumor efficacy, smaller tumor sizes, and apoptosis compared to systemically administered^18^. The aim of this study was, firstly, a new and innovative intra-tumoral drug delivery system composed of PEG–PLA–PEG triblock copolymer NPs in the form of nano-micelles was synthesized and investigated. It was used for the delivery of a line of three treatments (Cisplatin & 5-FU) as chemotherapeutic agents and (Cetuximab) as targeted anticancer therapy for the treatment of OSCC which was introduced in a mouse model. Secondly, comparing two routes of administration of the drug-loaded NPs, intra-tumoral and intravenous for the treatment of OSCC regarding histopathological evaluation at different time intervals.

## Methods

48 white mice (BALB/c mice) weighing 30 gm and 16 weeks old age were properly nourished, housed, induced, treated, and evaluated by a professional vet at El-mowasah Hospital, Animal Lab, Faculty of Medicine, Alexandria University. Evaluation for changes that occurred on the dorsum surface of the tongue after induction and treatment application. All mice were carefully inspected by magnification lens (X 20) daily and weighed weekly under anesthesia. Ethics committee approval was obtained from Mansoura University, Faculty of Dentistry (No. M03091019). The study was reported following ARRIVE guidelines. Scarification was held after the delivery of the drugs physically by cervical dislocation inconsistent with the American Veterinary Medical Association (AVMA) Guidelines for the Euthanasia of Animals (2020). All methods were performed following the relevant guidelines and regulations.

### Induction of OSCC (Chemical Carcinogenesis)

A water-soluble synthesized chemical carcinogen, 4-nitroquinoline 1-oxide (4-NQO) (98% pure, CAS no. 5657–5, Wako Pure Chemical Ind., Osaka, Japan) was used to induce OSCC chemically on the dorsum surface of the tongue. Concentrated ascending doses of 4-NQO 30, 50, 70, 90, 100, and 250 mg/ml were applied to the mice. Fresh solutions were prepared in each session. The tongue was stroked once with a no. 3 camel hairbrushes which had been dipped in the 4-NQO solution. Brushing was done day after day for 9 months. During chemical induction, all mice were anesthetized by inhalation of halothane vapor (1% isoflurane, Baxter, Belgium) in 100% oxygen using an animal anesthetizing evaporator (Minerva, Ester nay, France).

After 20 weeks from the start of induction, a sample of mice was selected (n = 2) which showed changes on the dorsum surface of the tongue after clinical examination with a magnification lens. Then a punch biopsy was obtained, frozen at 80 °C in a solution of fetal bovine serum (50%), and prepared for histopathological evaluation. Subsequently, there were other groups of selected mice biopsy according to the appearance of other changes on the dorsum surface of the tongue at 24 weeks (N = 2), 28 weeks (N = 16), 32 weeks (N = 24), and 36 weeks (N = 4) Table 1.

**Table 1:** Time of occurrence following the start of induction, grades, number of mice for control and test groups.

Time of occurrence following the start of induction	Grades	Number of mice for control group	Number of mice for test group	Chi-Square/Fischer exact test
20 weeks	Dysplasia	2 (8.3%)	0	FET = 2.08
				*P* = 0.489
24 weeks	Carcinoma in situ	2 (8.3%)	0	FET = 2.08
				*P* = 0.489
28 weeks	Grade I	8 (33.3%)	8 (33.3%)	X^2^ = 0.0
				*P* = 1.0
32 weeks	Grade II	10 (41.7%)	14 (58.3%)	X^2^ = 1.33
				*P* = 0.248
36 weeks	Grade III	2 (8.3%)	2 (8.3%)	FET = 0.0
				*P* = 1.0

It demonstrates non-statistically significant difference between mice in control and test groups. Among control group; 41.7% grade 2, 33.3% grade I, 8.3% dysplasia, carcinoma in situ & grade 3 versus 58.3% of test group are grade 2, 33.3% grade I and 8.3% grade 3.

### Histopathologic examination

All prepared histological sections were photographed using a digital camera (MICW16) installed on a light microscope (MEIJI MX5200L). All mice developed OSCC, and the following tissue changes were classified into dysplasia in the form of basilar hyperplasia (8.3 %), carcinoma in-situ (8.3 %). Several mice developed invasive Grade I OSCC (33.3 %), grade II OSCC was detected (41.7 %), and some developed Grade III OSCC (8.3 %), with increased signs of malignancy at the end of the induction period Table 1. There were no mice that succumbed or were lost during the study.

### Preparation and characterization of drug carrier

Acyl halide-terminated PLA (PLA-diCOCl) prepolymer was dissolved in anhydrous methylene chloride and one molar ratio of PEG was introduced into the solution. The molar ratio of the PLA prepolymer to PEG was 1:2. Anhydrous pyridine was added dropwise while the temperature was maintained at 0 °C. The reaction was carried out at room temperature for 12 h. The produced mixture was precipitated into hexane, washed 3 successive times with hexane/methylene chloride, and dried in vacum at room temperature for 24 h^19^. PEG–PLA–PEG copolymer was characterized by Fourier transform infrared spectroscopy (FTIR). FTIR spectra (Thermo Scientific Nicolet iS10 FT-IR KBr) were explored using UV/Vis absorption spectra in the region of 200,600 nm (Shimadzu 1700 Spectrometer). The chemical structure, composition, and average molecular weight (Mw) were measured for the synthesized copolymer. It was performed by using chloroform as a solvent and tetramethyl silane as an internal reference. The integral of PLA block peaks (CH–) and PEG blocks (–OCH_2_CH_2_) were used for determining the molar ratio of LA/EG (the ratio of PLA to PEG chain length) and then the calculation of Mw.

### Nano-micelle formation, characterization, and drug loading

The drug-free nano-micelles were synthesized from the prepared PEG–PLA–PEG copolymer by using the emulsification solvent evaporation method. Nano-micelles were obtained after freeze dehydration in the form of precipitated particles^19^. The micelles were examined using Scanning electron microscopy (Hitachi S-4700 Field Emission SEM, Hitachi High-Technologies Canada, Inc). SEM was used to directly observe the structures of nanoparticles and to examine the micelle morphology and particle size. The surface morphology of freeze-dried copolymer was evaluated by SEM where the copolymer particles were sputter-coated with a layer of gold.

### Drug loading

The PEG–PLA–PEG drug-loaded NPs were fabricated by the water-in-oil-in-water (W/O/W) solvent evaporation method. Briefly, a uniform solution was formed by dissolving 10 mg of PEG–PLA–PEG drug-free nano-micelles in 1 mL of trichloromethane and stirring at room temperature. The uniform solution with a dispersion consisting of 2 mg of the drug in 0.2 mL of Millipore water was sonicated at 200 W for 5 min to generate the W/O primary solution. The primary solution was centrifuged at 3000 rpm to remove free unreacted materials and emulsified with 2 mL of 2% polyvinyl alcohol solution containing Tween 80 as a surfactant then rotating at 9500 rpm for 30 min to generate the final W/O/W emulsion. Finally, the solution was evaporated to volatile trichloromethane at 30 °C for 30 min ^19^.

### Drug release profile

50 mg weighted NPs were shaken with 0.1 M deionized water at 37 ± 0.5 °C with slight agitation. The swollen NPs were removed at intervals from the buffer solution, blotted to remove excess liquid, and weighted on an electronic balance. A similar methodology was repeated for each drug for drug loading and drug release profile. The released amount of drug loaded was measured using a UV/Vis Spectrometer at a wavelength of 265 nm. Time dependence of the released amount of the drug was plotted for each type.

### Intra-tumoral delivery of the loaded micelles

36 weeks after the start of induction, all punch biopsies obtained revealed the appearance of all grades of dysplasia, carcinoma in situ, and invasive OSCC. Treatments were administered by an injection of loaded nano-micelles suspended in saline solution for both the control and test groups. Half of the mice (n = 24) were treated by systemic intravenous administration of cetuximab, cisplatin, and 5-FU-loaded micelles for the control group. The period of treatment application and continuation was 5 days. On day one, 0.12 mg/ml cisplatin was injected first, and after an hour, 0.06 mg/ml cetuximab was injected. From day two till day five, 0.0144 mg/ml of 5-FU was injected. A fresh aliquot was prepared for each session every day through 5 days of administration.

With equivalent doses and the same protocol followed for the control group, the other half of the mice (n = 24) were treated by intralesional injections of cetuximab, cisplatin, and 5-FU-loaded micelles for the test group. All lesion sites were carefully inspected by magnification lens (X 20) daily and mice were weighed weekly under anesthesia till scarification time, one month following the last dosage applied for treatment.

### Histopathologic examination

After scarification, a biopsy was obtained. All prepared histological sections were photographed using a digital camera, as mentioned before, re-evaluated after treatment application, and compared with after-induction biopsies. Regarding the control group, the following tissue changes after treatment application were normal tissue and grade I with a percentage of 8.3%, 41.7% carcinoma in situ, 25% dysplasia, and necrosis 16.7%. For the test group, 50% were normal tissue, and the other half showed necrosis Table 2.

**Table 2:** Grades of OSCC after treatment, number of mice for control and test groups.

Grades after Treatment	Number of mice for control group	Number of mice for test group	Chi-Square/Fischer exact test
Normal	2 (8.3%)	12 (50%)	X^2^ = 10.08
			*P* = 0.001*
Necrosis	4 (16.7%)	12 (50%)	X^2^ = 6.0
			*P* = 0.01*
Dysplasia	6 (25%)	0	X^2^ = 6.86
			*P* = 0.008*
Carcinoma In situ	10 (41.7%)	0	X^2^ = 12.63
			*P* = 0.0003*
Grade I	2 (8.3%)	0	X^2FET^ = 2.08
			*P* = 0.489

It illustrates statistically significant difference of Grades after treatment between test and control groups. Among control group; 41.7% carcinoma in situ, 25% dysplasia, 16.7% necrosis, 8.3% grade I and normal. For test group; 50% are normal and 50% shows necrosis.

#### Statistical analysis and data interpretation

Data analysis was performed by using computer software. Qualitative data were described using numbers and percentages. Chi-Square and Fischer exact tests were used to compare tumor grade before and after treatment for each test group (n-28, ά = 0.05) Table 3.

**Table 3:** Grade (percentage), time, cross tabulation, and their significance for OSCC induced in mice.

Time		Grades							Pearson Chi- Square
		Normal	Necrosis	Dysplasia	Carcinoma in situ	Grade I	Grade II	Grade III	
Start	Intravenous			2 (8.3)	2 (8.3)	8 (33.3)	10 (41.7)	2 (8.3)	X^2^ = 4.67 *P* = 0.323
	Intralesional			0	0	8 (33.3)	14 (58.3)	2 (8.3)	
	Total			2	2	16	24	4	
Final	Intravenous	2 (8.3)	4 (16.7)	6 (25)	10 (41.7)	2 (8.3)			X^2^ = 29.14 *P* < 0.001*
	Intralesional	12 (50)	12 (50)	0	0	0			
	Total	14	16	6	10	2			48

It shows statistically significant difference between different grades of OSCC at final assessment with 50% of intralesional have normal and 50% necrosis. However higher grades are detected for intravenous with 41.7% of intravenous are carcinoma in situ, 25% dysplasia, 16.7% necrosis, and 8.3% grade 1.

## Results

### FTIR characterization of PLA, PEG prepolymers, and triblock copolymer micelles

**Figure 1:**
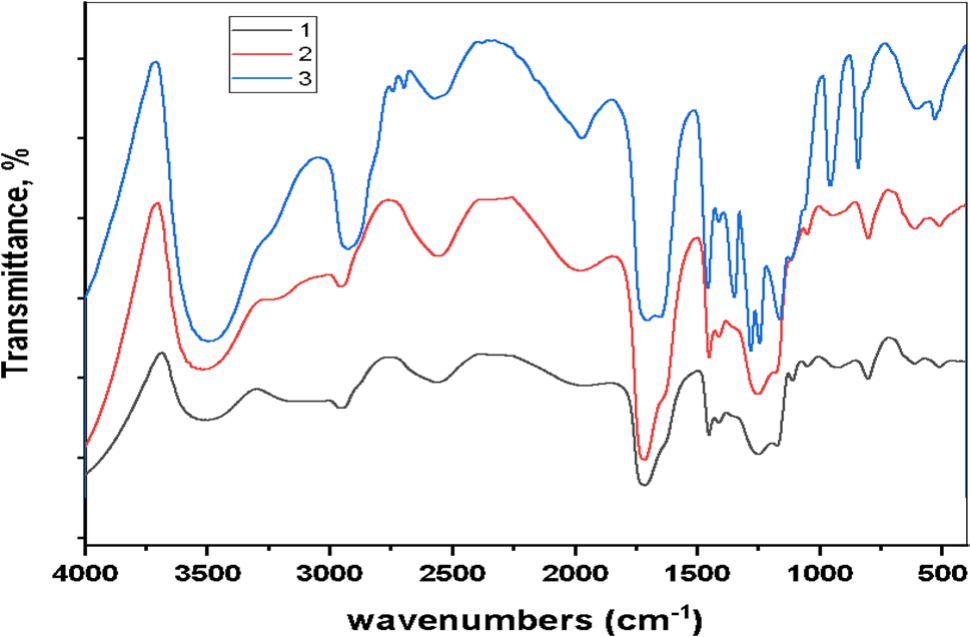
Fourier transform infrared spectroscopy (FTIR) Spectra of two prepolymers and copolymerized triblock polymers. (**1**). (Black color) FTIR Spectrum of PLA prepolymer (**2**)**.** (Red color) FTIR Spectrum of PEG–PLA–PEG triblock copolymer (**3**). (Blue color) PEG prepolymer FTIR analysis.

Figure 1 showed three different FTIR spectra, FTIR analysis of PLA prepolymer showed a wave band at 3499 cm^−1^ which was corresponding to the O–H stretching vibration of -COOH end groups, and the bands at 2957 and 2455 cm^−1^ were due to the C–H stretching vibration of –CH_3_ and –CH– groups. The sharp peak at 1713 cm^−1^ was assigned to the stretching vibration of the ester carbonyl group (Fig. 11) (black color). The FTIR spectra of the PEG-PLA- PEG triblock copolymer was shown in (Fig. 12) (red color) showing all typical absorbing bands of PLA and PEG prepolymer suggesting the presence of the two-component blocks in the copolymer. For PEG prepolymer^20^, FTIR analysis showed the band at 3448 cm^−1^ characteristics to the O–H stretching vibration of –COOH end groups and the bands at 2855 cm^−1^ were due to the C–H stretching vibration of –CH_3_ and –CH– groups. The sharp peak at 1112 cm^−1^ was assigned to the stretching vibration of the –C–O–C– group as shown in (Fig. 13) (blue color).

### Nano-micelle characterization, and drug loading

**Figure 2:**
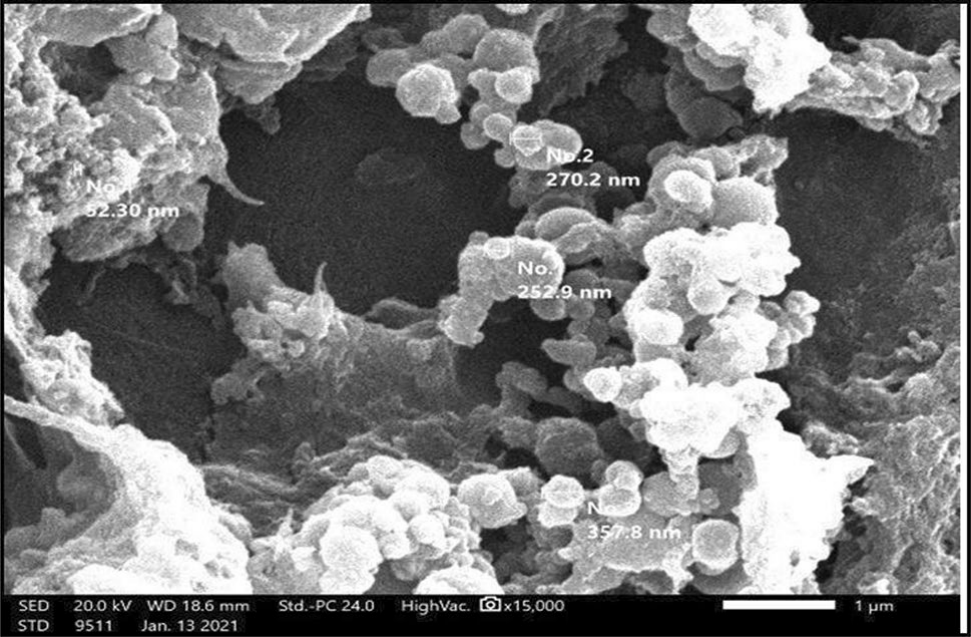
Scanning electron microscopy (SEM) photomicrograph of synthesized PEG–PLA–PEG triblock copolymers with magnification power (X 60,000). The samples were observed by JSM-IT200InTouchScopeTM [JOEL Benelux-supplier of Electron Microscopes (Akishima, Tokyo)].

SEM imaging of triblock copolymer micelles revealed a regular spherical structure with smooth and uniform surfaces without signs of collapse or agglomeration. It was noticeable that the micelles were varying in size and were constructed from small copolymer clusters; however, the average size of the triblock copolymer particles was in the range of 60–201 nm **(**Fig. 2**).**

**Figure 3:**
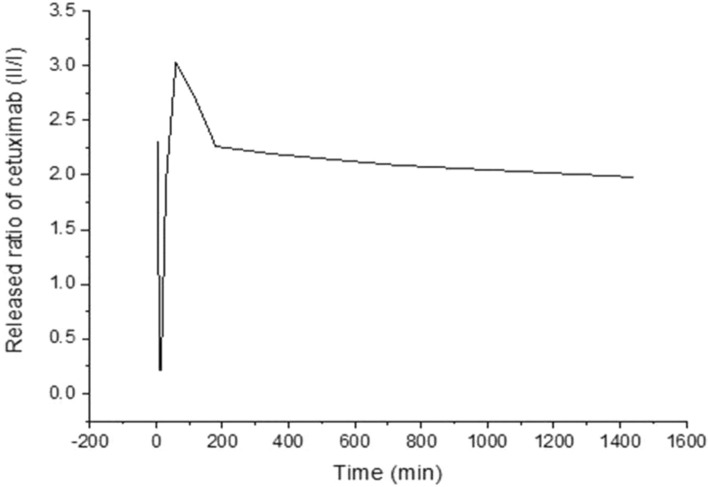
Release curve of cetuximab loaded PEG–PLA–PEG nano-micelles at room temperature and neutral PH.

**Figure 4:**
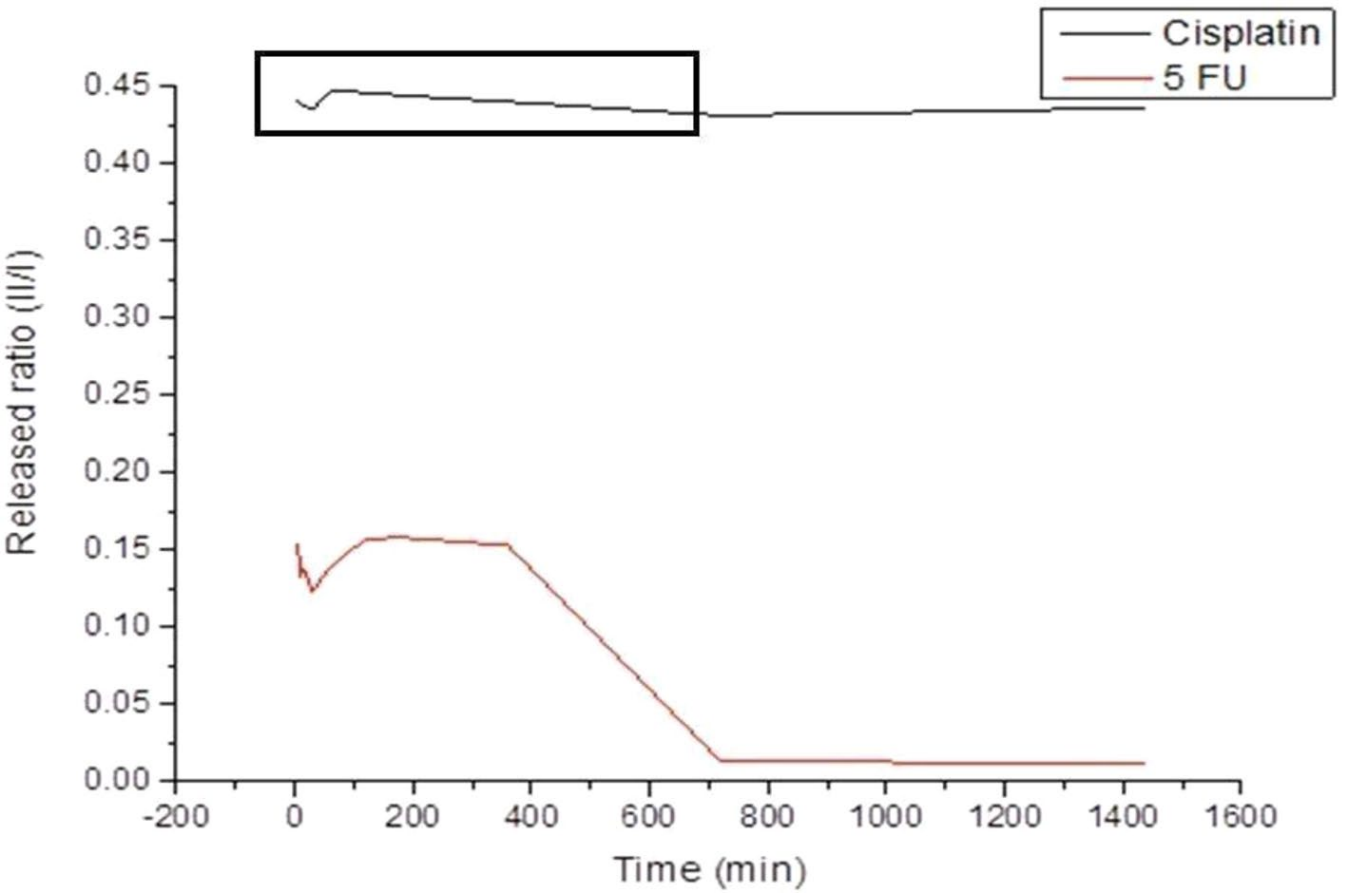
Release curves of cisplatin (black color) and 5-FU (red color) loaded PEG–PLA–PEG nano-micelles at room temperature and neutral PH.

For cetuximab, the release profile indicated a high initial burst of about 3% of the total mass for up to 2 h followed by a gradual decrease in the release for another two hours. After a long time up to 26 h, a slow and steady phase of release was observed, Fig. 3**.** The behavior of the 5-FU release profile is divided into four phases first, small initial bursts for about two hours. Hence, its concentration in the dissolution medium remained constant for up to 4 h. Another 4 h phase of a slow gradual decrease followed. Finally, it took about 12 h for the small amount of the drug to be released constantly, Fig. 4. Cisplatin, first, showed an abrupt initial rapid release. On the other hand, in the second phase of drug release, it was noticed that there was a sudden long and slow decrease in the release rate extended for about 10 h. The third phase showing an increased release lasts for nearly 7 h. Release kinetics are summarized in Table 4.

**Table 4:** Released ratio of cetuximab, cisplatin and 5-Fu loaded nano-micelles at each given time in minutes.

Time (Min)	5	10	15	30	60	120	180	360	720	1440
Released ratio = II/I of cetuximab	2.310	0.215	0.215	1.930	3.030	2.690	2.260	2.190	2.090	1.980
Released ratio = II/I of cisplatin	0.440	0.438	0.438	0.435	0.447	0.446	0.444	0.440	0.431	0.436
Released ratio = II/I of 5-FU	0.154	0.133	0.138	0.123	0.139	0.157	0.158	0.153	0.013	0.012

### Histopathologic examination

**Figure 5:**
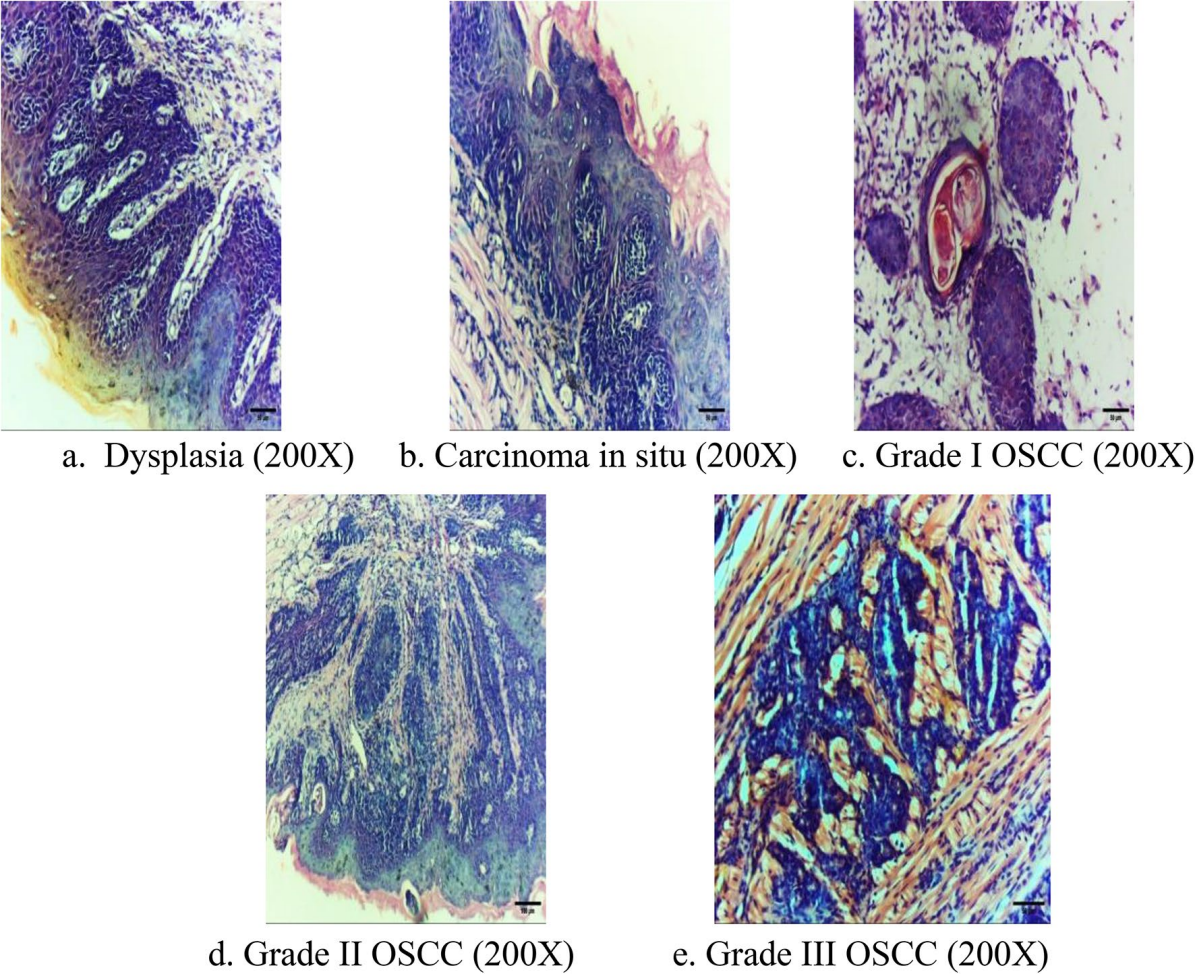
(**a**)–(**e**) Photomicrographs for histopathological examination of the Hematoxylin & Eosin-stained tongue specimen after induction of OSCC, at week 20, the induction site started to develop epithelial dysplasia (200X) (**a**). Four weeks later, carcinoma in situ (200X) started to appear on the dorsum surface of the tongue (**b**). Several mice developed invasive OSCC with some being Grade I (200X) (**c**) at week 28. Others yield Grade II (200X) (**d**) at week 32, and some mice developed Grade III (200X) (**e**) at the end of the induction period after 36 weeks.

Histopathological evaluation of the prepared Hematoxylin & Eosin punch biopsies revealed that 20 weeks after induction, tissue started to develop epithelial dysplasia in the form of basilar hyperplasia (BH), abnormal mitosis, loss of cellular adhesion (LE), and abnormal keratinization (AK) that did not extend beyond the epithelial two-thirds thickness **(**Fig. 5a**)**. Four weeks later, carcinoma in situ started to appear on the dorsum surface of the tongue by extension of dysplastic changes to the full epithelial thickness with intact basement membrane Fig. 5b. Several mice developed invasive Grade I OSCC **(**Fig. 5c**)** after 28 weeks with cell nests (CN) showing dysplastic changes and epithelial pearls (EP). Grade II OSCC was detected after 32 weeks with cell nests only and no keratin formation Fig. 5d. Some mice developed Grade III OSCC, after 36 weeks, with increased signs of malignancy at the end of the induction period with dysplastic epithelial cells forming sheets (ES) and no nests or keratin Fig. 5e.

**Figure 6:**
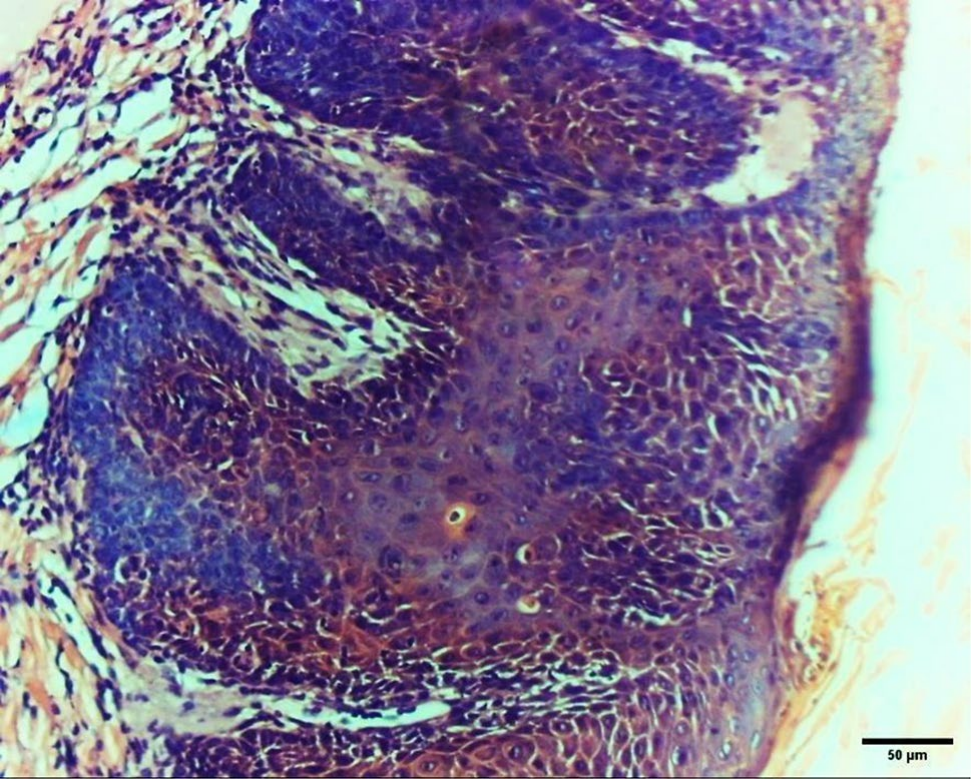
Photomicrograph for histopathological examination of the tongue specimen after application of systemic drug-loaded NPs for the control group showing carcinoma in situ (200X).

**Figure 7:**
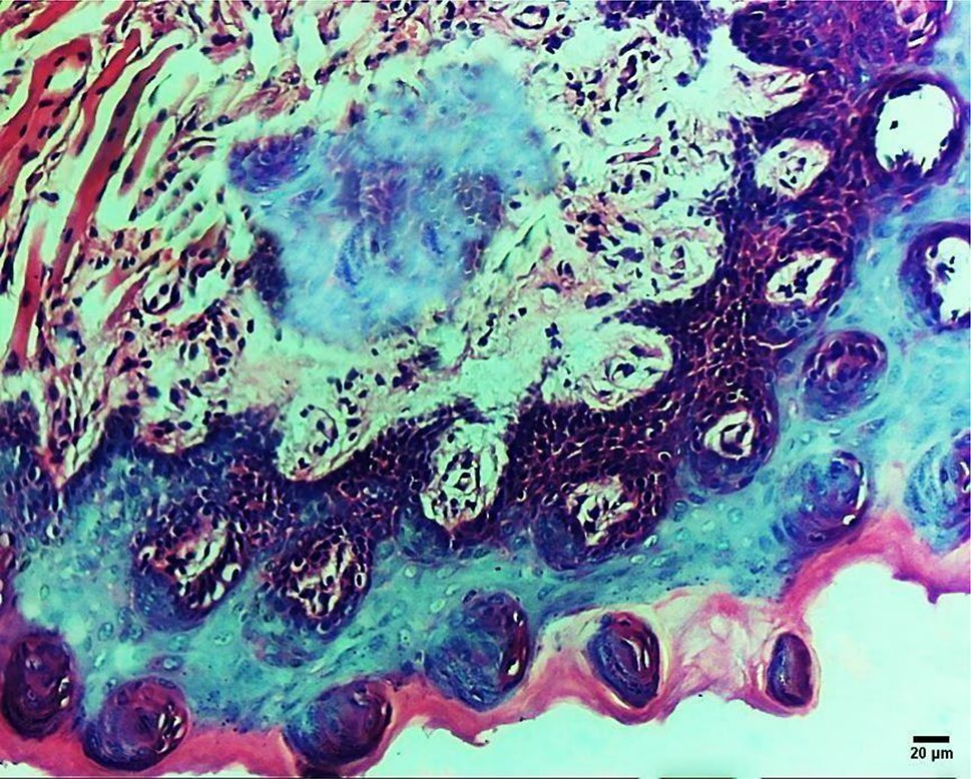
Photomicrograph for histopathological examination of the tongue specimen after application of intra-tumoral drug-loaded NPs for test group showing necrosis (200X) of the tissue in 50% of total specimen.

Systemic administration of NPs reduced the degree of dysplasia in the surface epithelium as it changed dysplastic epithelium into normal and carcinoma in situ into necrotic epithelium (NE) with loss of cellular architecture and ballooning with no evident dysplasia. It also reduced the malignancy of the tested groups by converting grade I tumors into epithelial dysplasia and grade II tumors into carcinoma in situ with the elimination of the invasive element of the tumor (Fig. 6) in the form of basilar hyperplasia (BH), abnormal mitosis, loss of cellular adhesion (LE), and abnormal keratinization (AK) extending to the full thickness of the epithelium. Others converted grade III to grade I as the dysplastic epithelial cells started to form cell nests and epithelial pearls instead of sheets. However, intra-tumoral application of NPs resulted in significantly better results (X^2^ = 29.14, *P* < 0.001) by eliminating the invasive elements in grade I and II malignancy and restoring the epithelium to its normal condition and induced necrosis in the invading elements of grade III malignancy preventing its expansion to deeper tissues, Fig. 7. The study was designed to evaluate the intra-tumoral drug delivery system composed of PEG–PLA–PEG triblock copolymer NPs in the form of nano-micelles after searching in the published evidence it had been found that the common route of application was systematic which had many drawbacks overcome by targeting with intra-tumoral application and this was top listed in the list of the study objectives.

## Discussion

Dealing with cancer is a challenge because of nonselective damage caused by therapeutic agents. The application of nanotechnology-based carriers as polymeric nano-micelles offered solutions to these restrictions by local delivery of chemotherapeutics directly to the cancer cells. Although these systems are very small if compared to the cell size, they have a great surface area to carry numerous functional moieties for targeting the cell surface^21^. The new attention for drug delivery approaches in oral cancer treatment is intralesional or local drug delivery of loaded NPs^22^. These injectable nano-micelles have numerous superior properties such as cytocompatibility, promising mechanical properties, favorable permeability, injectability, controllable degradability, and the ability to target specific cells. In addition, their ability for enhancing the stability and solubility of poorly soluble drugs^23^.

PLA degradable-based polymers have unique characteristics for the exploitation of NPs for the delivery of hydrophobic drugs. These NPs are widely used compared to non-degradable particles and demonstrated massive translational potential in a wide range of clinical applications^24^. When PLA is copolymerized with highly hydrophilic, flexible, and bio-inert polymers like PEG which formed the outer corona of triblock, it can be administered successfully for the delivery of chemotherapeutic drugs in vivo^25,26^.

In agreement with the current study results, in 2020, Nairrita concluded that PEG was the polymer of choice and approved by the FDA for clinical applications mainly because of its biocompatibility, molecular weight (1–15 kDa) that provides an optimum residence time in the body, denser water-soluble outer corona, and stealth characteristics to the micelle against attaching immune cells^25^. Molecules that targeted the epidermal growth factor receptor (EGFR) have favorable results in the prognosis of OSCC. Cetuximab as a monoclonal antibody and EGFR inhibitor was approved by FDA in February 2004^27^. PEG–PLA–PEG NPs have been selected in the current study as a delivery system. It was applied for targeted, intralesional, and intravenous delivery of the selected drugs with controlled and sustained release. Hence, reducing the need for high dosages of systemic intravenous injections.

SEM photomicrographs illustrate that the nano-micelles vary in size and are constructed from small copolymer clusters (with an average diameter of 60–300 nm in size) which may not only escape biliary excretion and renal filtration but can also accumulate in the tumor site. Sizes larger than 400 nm may be trapped in the reticuloendothelial system or the hepatic clearing system^28^. FTIR peaks indicate that all prementioned typical absorbing bands of PLA and PEG prepolymer were detected suggesting the presence of the two-component blocks in the copolymer. Moreover, the bands of –OH end groups are much smaller than those of the two macromonomers, indicating that most of the end groups are participating in the condensation reaction and producing a much higher molar mass of the triblock copolymer^20^.

For better therapeutic results, the current drug release profile suggests higher drug accumulation in the cancer cells. These characteristics result in lowering the drug dose and decreasing the risk of non-cancerous tissue toxicity. Therefore, polymeric nano-micelles are efficient in cancer cell penetration, enhancing site-specific delivery of selected therapeutics^29^. The drug release from NPs was measured under constant physiological conditions (neutral pH and room temperature). The reason for variations in release profiles could be related to the different hydrolysis-induced degradation rates of the polymers, proteins, and other factors^30^.

The chemical structure of the PEG–PLA–PEG tri-block copolymer showed the presence of C–O–C, C=O, and OH functionalities. Such functional groups assist the polymeric chains to be packed close to each other under the effect of hydrogen bonding formed between the terminal OH groups and/or between OH and C–O–C bonds within the polymer matrix. After loading the drug into the polymer matrix, the drug is distributed through the matrix and between the chains. The drug molecules are also attracted to the polymer chains through the hydrogen bonding between the OH of the polymer chains and NH of 5-FU and N and O in cetuximab. On the other hand, the polymer can form electrostatic attraction between C=O and C-F bonds in 5-FU, the bulk molecule of cisplatin, and the polar C-O and C-N bonds in cetuximab^31,32^.

For the cetuximab release profile, the high initial burst release may be attributed to the hydration of surface layers of the nano-micelles leading to the quick release of the surface portion of the drugs^33^. By this time, it shows a gradual decrease in the release profile due to the poor solubility of PLA and the drug in the releasing media^34,35^. The steady third phase of release is the result of two opposite factors. Firstly, long contact of the nano-micelles with the dissolution medium exhibits disintegration of the physical interactions linking the polymer chains. Secondly, resistance to water penetration is manifested by poor soluble drugs as explained before^34^.

The Nonspecific and incomplete release was also discussed by Mallery SR et al., one of the explanation theories was non-specific adsorption and incorporation of some proteins to the hydrophobic core of NPs^36^. The first and final phases in the release profile for 5-FU may be attributed to the same prementioned explanation for the initial burst and steady release in cetuximab. Hence, the constant concentration up to 4 h, may be due to the resistance resulting from strong hydrogen bonding between different chains of polymer matrices. The slow gradual decrease of release in the third phase can be explained by the complex formation between the positively charged drug and the negatively charged polymer matrix^37^.

Regarding cisplatin. Firstly, the abrupt initial rapid release is related to proteins located on the surface or near the surface of the NPs in the channels forming the linkage between the internal droplets and external aqueous phase established during the organic solvent removal step and solidified at the process of nano-micelles hardening^38^. On the other hand, the abrupt slow decrease in the release rate in the second phase of drug release is related to the encapsulated proteins that are released through both gradual degradation and diffusion of the hydrophilic PEG matrix due to hydrolysis in water. The third phase of the release profile showing an increased release of the drug seemed to be caused by enhancing the ratio of hydrophilic PEG to the hydrophobic sequence of PLA which has increased the water diffusion to the system, polymer disintegration, and rapid drug release^34^. The selection of a mouse model was due to its ability to form all phases of OSCC (dysplasia, carcinoma in situ, and OSSC with its different grades) and its sensitivity to demonstrating the effects of anti-cancer treatments^39,40^. Carcinogenesis is a long multistage process in which invasion was induced after several months but it is proven by some studies that it was hard to induce OSCC with other chemicals^41–43^. Punch biopsies confirmed the formation of different stages of OSCC^44^. The anticancer effect against tumor growth in OSCC-induced mice treated with cisplatin-loaded NPs was 4.4–6.6-fold greater than the control group. Additionally, the controlled release of cisplatin from these NPs induced apoptosis and resulted in lowered neurotoxicity and nephrotoxicity in contrast to the administration of cisplatin in solution^45^. Intra-tumoral injections resulted in massive degeneration of the dysplastic changes that occurred in the surface epithelium while other specimens showed signs of necrosis with no evident dysplasia. In addition, exfoliation of nests within the cells and epithelial pearls instead of sheets was also observed as the neoplastic cells started to form as a sign of a reduction in malignancy.

The limitations of the current study are that it is still based on synthetic treatments for the eradication of cancer types instead of natural extracts. In addition, the lengthy multistep process is followed for the induction of cancer. Hence, these factors were beneficial to be considered when selecting the type, and dose of chemical carcinogen and the treatments used. Lastly, the future directions of the current study can be summarized as follow:

- The PEG–PLA–PEG nano-micelles still need to be carefully studied in several animal models and eventually in human patients.
- The in vivo tumor accumulation profiles of drug-loaded PEG–PLA–PEG nanoparticles can be clearly visualized by monitoring the whole-body fluorescent intensity in chemically induced carcinogenesis mice models.
- Furthermore, pre-clinical animal studies need to be moved forward for developing clinical trial approaches to further enhance human health.
- Further research will be helpful for early diagnosis or prevention of OSCC through efforts for cessation of predisposing factors. In addition, biomarkers must be studied to predict the behavior of tumor cells and used to characterize tumors.

## Conclusions

Based on the results and within the limitations of this study, it is concluded that using PEG–PLA–PEG tri-block copolymer as a drug delivery system and adjusting the content and Mw of LA to EG and the PEG–PLA ratio increases the drug loading of hydrophobic drugs, reduces particle sizes, and prolongs blood circulation time. In addition, using synthesized PEG–PLA–PEG nano-micelles as a delivery system for intra-tumoral injection of targeted anticancer agents (Cetuximab) and Cisplatin & 5-FU, chemotherapeutic agents, as a new approach is considered a promising route for eradication of OSCC which also offer an improved response of OSCC to selected therapeutic agents.

## Data Availability

The datasets used and/or analyzed during the current study are available from the corresponding author upon reasonable request.
